# Supplementary material for: Repeat induces not only gene silencing, but also gene activation in mammalian cells
Source: PLoS One. 2020 Jun 24;15(6):e0235127. doi: 10.1371/journal.pone.0235127 (PMC7313748; doi:10.1371/journal.pone.0235127)
Supplement: S1 Fig — (PPTX) [file pone.0235127.s001.pptx]

## Slide 1
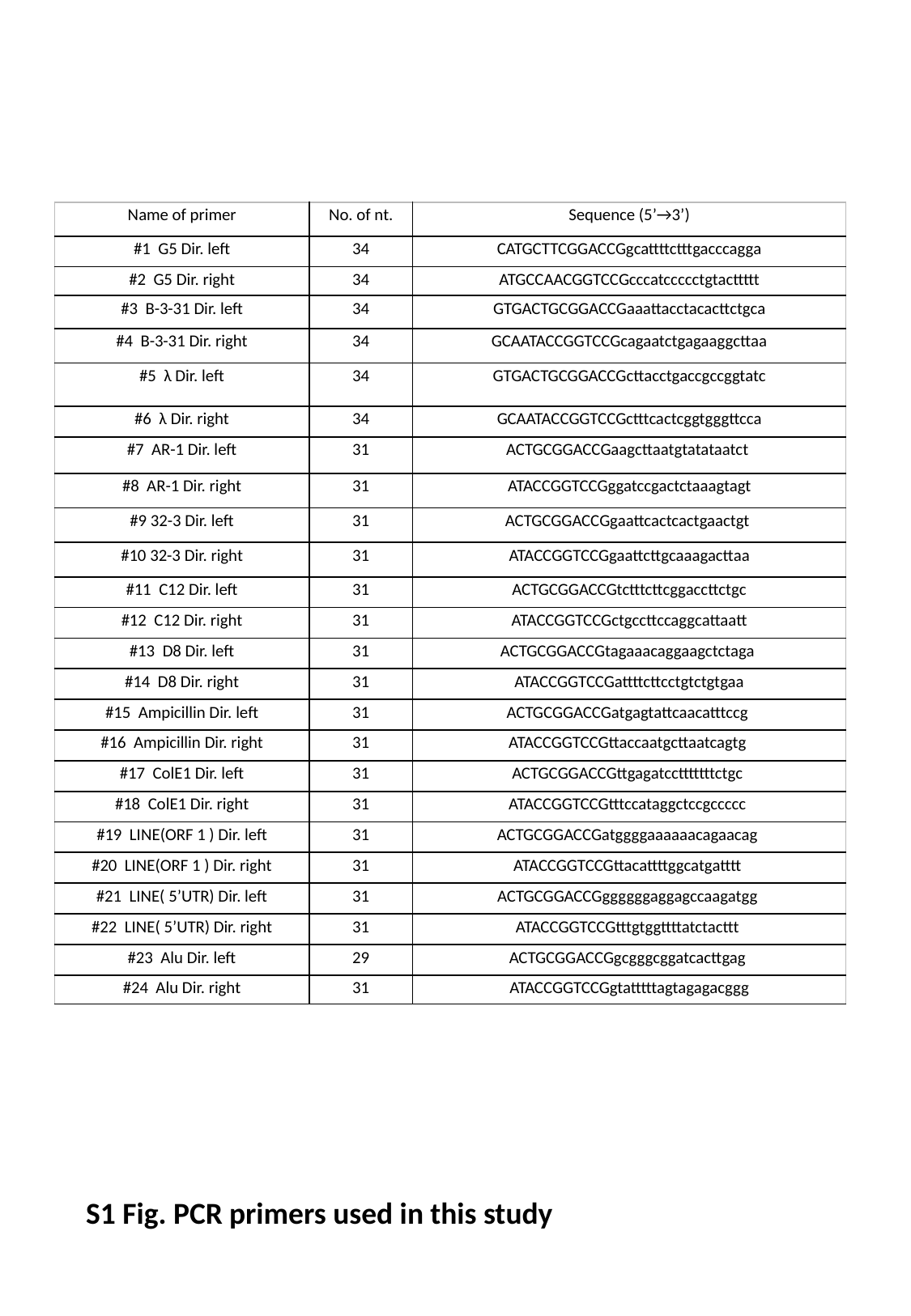

| Name of primer | No. of nt. | Sequence (5’→3’) |
| --- | --- | --- |
| #1 G5 Dir. left | 34 | CATGCTTCGGACCGgcattttctttgacccagga |
| #2 G5 Dir. right | 34 | ATGCCAACGGTCCGcccatccccctgtacttttt |
| #3 B-3-31 Dir. left | 34 | GTGACTGCGGACCGaaattacctacacttctgca |
| #4 B-3-31 Dir. right | 34 | GCAATACCGGTCCGcagaatctgagaaggcttaa |
| #5 λ Dir. left | 34 | GTGACTGCGGACCGcttacctgaccgccggtatc |
| #6 λ Dir. right | 34 | GCAATACCGGTCCGctttcactcggtgggttcca |
| #7 AR-1 Dir. left | 31 | ACTGCGGACCGaagcttaatgtatataatct |
| #8 AR-1 Dir. right | 31 | ATACCGGTCCGggatccgactctaaagtagt |
| #9 32-3 Dir. left | 31 | ACTGCGGACCGgaattcactcactgaactgt |
| #10 32-3 Dir. right | 31 | ATACCGGTCCGgaattcttgcaaagacttaa |
| #11 C12 Dir. left | 31 | ACTGCGGACCGtctttcttcggaccttctgc |
| #12 C12 Dir. right | 31 | ATACCGGTCCGctgccttccaggcattaatt |
| #13 D8 Dir. left | 31 | ACTGCGGACCGtagaaacaggaagctctaga |
| #14 D8 Dir. right | 31 | ATACCGGTCCGattttcttcctgtctgtgaa |
| #15 Ampicillin Dir. left | 31 | ACTGCGGACCGatgagtattcaacatttccg |
| #16 Ampicillin Dir. right | 31 | ATACCGGTCCGttaccaatgcttaatcagtg |
| #17 ColE1 Dir. left | 31 | ACTGCGGACCGttgagatcctttttttctgc |
| #18 ColE1 Dir. right | 31 | ATACCGGTCCGtttccataggctccgccccc |
| #19 LINE(ORF 1 ) Dir. left | 31 | ACTGCGGACCGatggggaaaaaacagaacag |
| #20 LINE(ORF 1 ) Dir. right | 31 | ATACCGGTCCGttacattttggcatgatttt |
| #21 LINE( 5’UTR) Dir. left | 31 | ACTGCGGACCGggggggaggagccaagatgg |
| #22 LINE( 5’UTR) Dir. right | 31 | ATACCGGTCCGtttgtggttttatctacttt |
| #23 Alu Dir. left | 29 | ACTGCGGACCGgcgggcggatcacttgag |
| #24 Alu Dir. right | 31 | ATACCGGTCCGgtatttttagtagagacggg |
S1 Fig. PCR primers used in this study
