# Supplementary material for: Repeat induces not only gene silencing, but also gene activation in mammalian cells
Source: PLoS One. 2020 Jun 24;15(6):e0235127. doi: 10.1371/journal.pone.0235127 (PMC7313748; doi:10.1371/journal.pone.0235127)
Supplement: S2 Fig — (PPTX) [file pone.0235127.s002.pptx]

## Slide 1
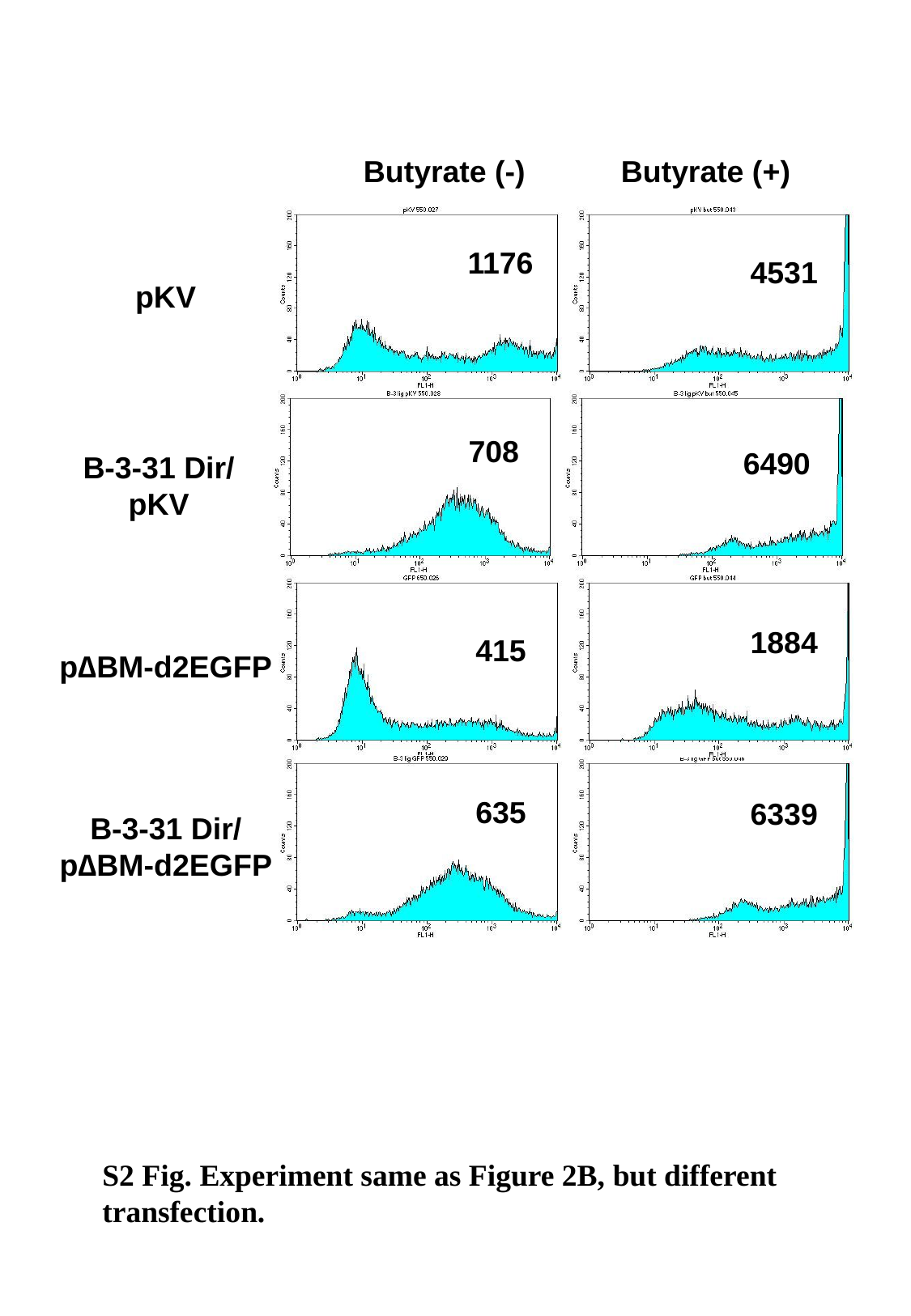

Butyrate (-)
Butyrate (+)
1176
4531
pKV
708
6490
B-3-31 Dir/
pKV
1884
415
p∆BM-d2EGFP
635
6339
B-3-31 Dir/
p∆BM-d2EGFP
S2 Fig. Experiment same as Figure 2B, but different transfection.
