# Supplementary material for: Repeat induces not only gene silencing, but also gene activation in mammalian cells
Source: PLoS One. 2020 Jun 24;15(6):e0235127. doi: 10.1371/journal.pone.0235127 (PMC7313748; doi:10.1371/journal.pone.0235127)
Supplement: S3 Fig — (PPTX) [file pone.0235127.s003.pptx]

## Slide 1
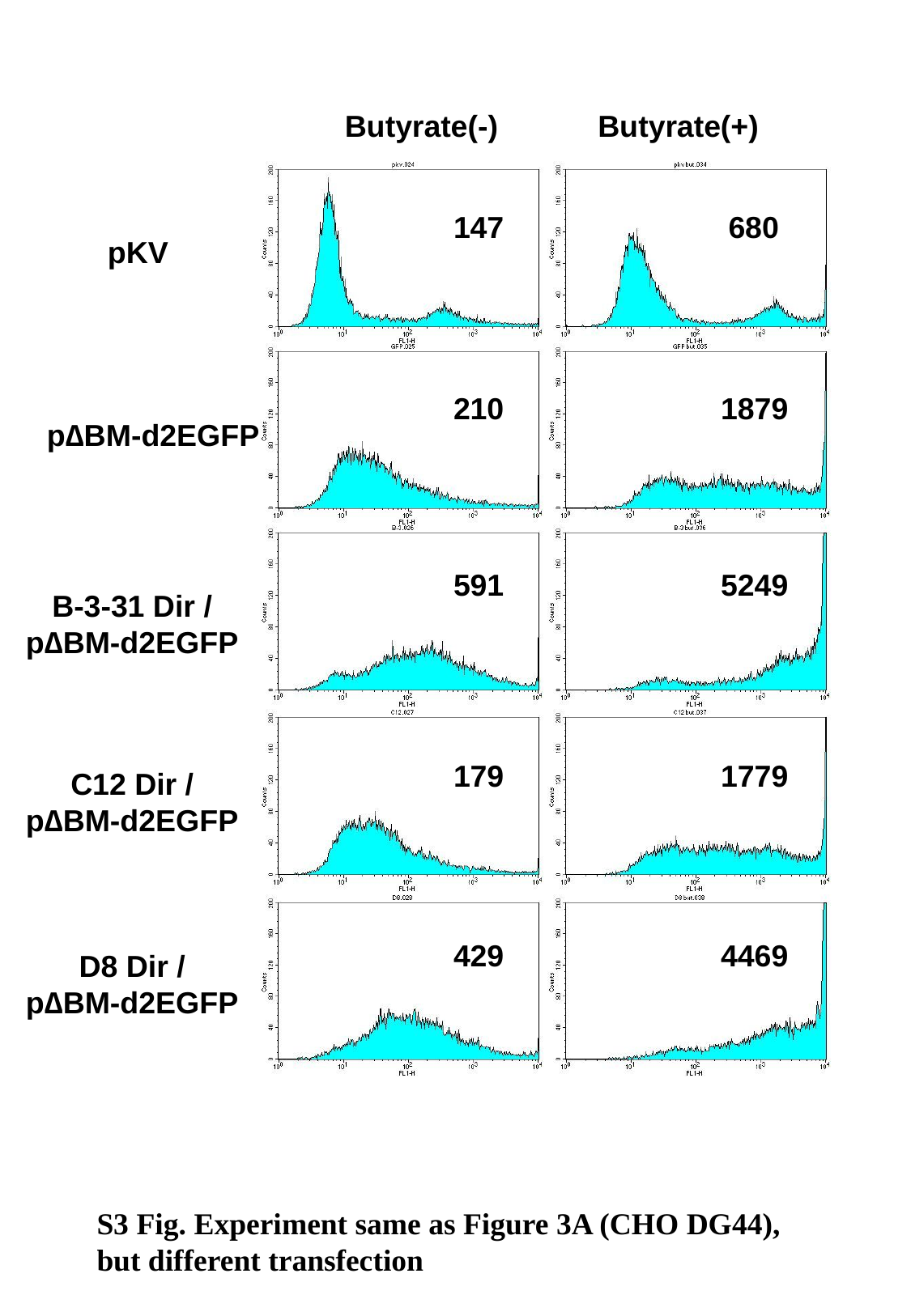

Butyrate(-)
Butyrate(+)
147
680
pKV
210
1879
p∆BM-d2EGFP
591
5249
B-3-31 Dir /
p∆BM-d2EGFP
179
1779
C12 Dir /
p∆BM-d2EGFP
429
4469
D8 Dir /
p∆BM-d2EGFP
S3 Fig. Experiment same as Figure 3A (CHO DG44), but different transfection
