# Supplementary material for: Repeat induces not only gene silencing, but also gene activation in mammalian cells
Source: PLoS One. 2020 Jun 24;15(6):e0235127. doi: 10.1371/journal.pone.0235127 (PMC7313748; doi:10.1371/journal.pone.0235127)
Supplement: S1 Raw images — (PDF) [file pone.0235127.s004.pdf]

All Images apper in Fig. 1D

1 2 3 4 5 6　　1 2 3 4 5 6

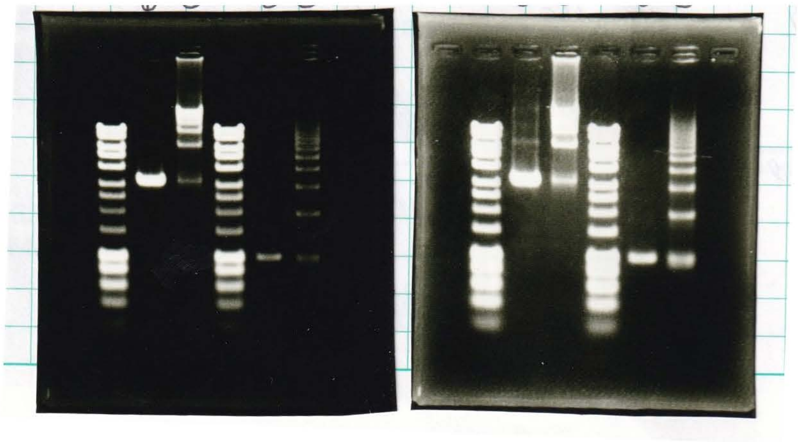

1, 4; Molecular weight marker

2; B-3-31 unligated

3; B-3-31 ligated

5; G5 unligated

6; G5 ligated

Right and left images were identical gel, and were taken at different conditions.

X 1 2 3 4 5 6 X

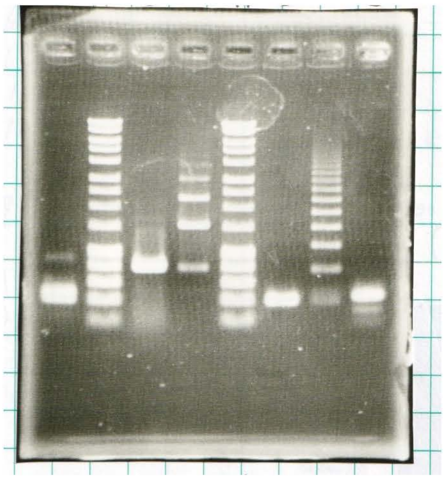

1, 4; Molecular weight marker

2; 32.3 unligated

3; 32-3 ligated

5; AR-1 unligated

6; AR-1 ligated

X X 1 2 3 X X X

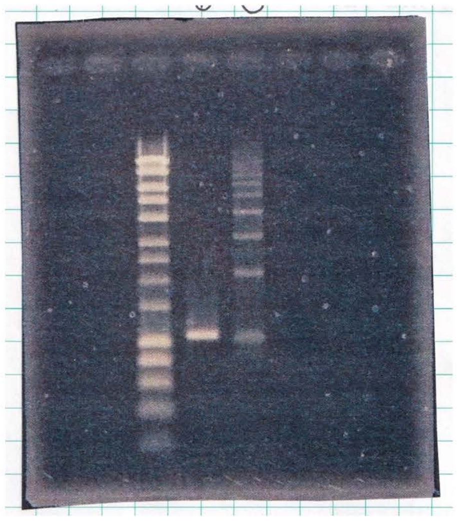

1; Molecular weight marker

2. lambda unligated

3. lambda ligated

X 1 2 3 4 5 6 X

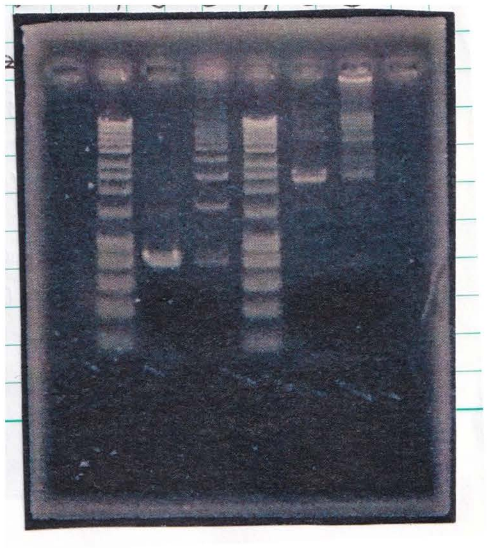

1, 4; Molecular weight marker

2; C12 unligated

3; C12 ligated

5; D8 unligated

6; D8 ligated

X 1 2 3 4 5 6 X

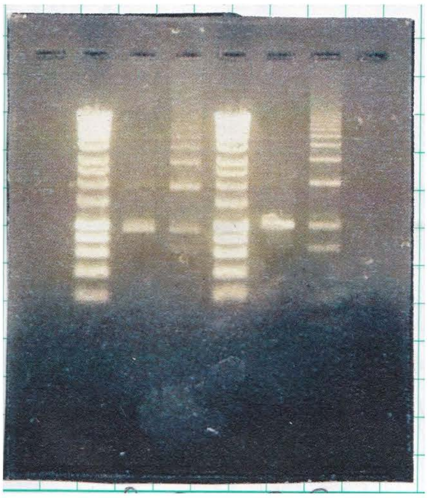

1, 4; Molecular Weight marker

2; LINE (5' UTR) unligated

3. LINE (5' UTR) ligated

5; LINE (ORF1) unligated

6; LINE (ORF1) ligated

X 1 2 3 4 5 X X

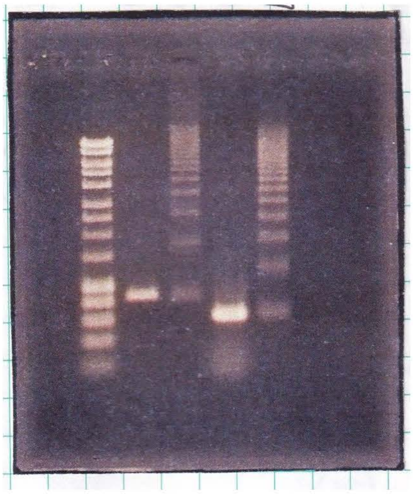

1; Molecular Weight marker

2; Amp unligated

3. Amp ligated

5; ColE1 unligated

6; ColE1 ligated

X 1 2 X 3 4 X X

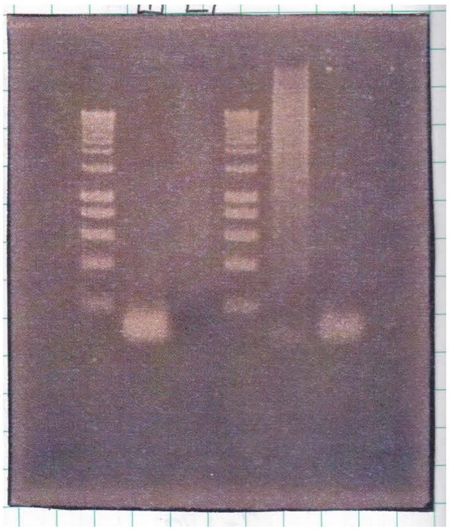

1, 3; Molecular Weight marker

2; Alu unligated

4. Alu ligated
