# Supplementary material for: Repeat induces not only gene silencing, but also gene activation in mammalian cells
Source: PLoS One. 2020 Jun 24;15(6):e0235127. doi: 10.1371/journal.pone.0235127 (PMC7313748; doi:10.1371/journal.pone.0235127)
Supplement: Attachment — Submitted filename: Response_to_Reviewers_.docx [file pone.0235127.s005.docx]

PONE-D-20-07143
Repeat induces not only gene silencing (RIGS), but also gene activation (RIGA) in mammalian cells
PLOS ONE

The following set of changes are required  to address the Reviewers' and AE's comments regarding textual clarity, statistical analysis and scientific rigor:
1. Textual Issues

- First, the manuscript should be proofread by another investigator familiar with journal publication, to eliminate grammatical errors that are present throughout.  Also please place line numbers in the text for easier textual reviewing.
- ------------------The original manuscript had already underwent English editing by a professional English editing service offered by Editage (Cactus communication co.). We can provide a certificate from that company. However, we sent the revised manuscript back for further English editing. We have included line numbers and formatted the manuscript in accordance with *PLOS ONE* formatting guidelines.
- Second, the manuscript is written for a specialty audience.
  - A) The information in the abstract and the manuscript should be accessible to a general audience, eliminating excess technical jargon.
  - -------------Thank you for your comments. We reviewed the abstract and the entire manuscript, and modified the text to be easily accessible to a general audience.
  - B) The introduction should provide more background regarding RIGS, a less technical description of the labs’ previous work, and an elimination of methods/results.
  - -----------We have added more background information regarding RIGS (ref 14, lines 61-69) in the revised manuscript. The minimum description regarding our previous works was required for explaining the background, specific techniques, and rationale for the methodology. Such background information originally appeared in the Introduction has been moved to a new section entitled “Experimental design to evaluate RIGS and RIGA” in Methods of the revised manuscript. We further reviewed and revised the manuscript for better clarity.
  - C) The Methods should be describe more fully and include statistical analysis.
  - ---------We have added the statistical analyses accordingly, which appear in the newly added Figure 5 and the relevant sections in the revised manuscript.
  - D) The Results must provide more introductory context (e.g, reason for treatment with butyrate), hypothesis to be tested, and a better textual description of the data presented in the Figures, and a simple conclusion from the data shown.
  - -----------The reason behind the butyrate treatment was explained in the Methods section of the original manuscript. We have moved the explanation for the butyrate treatment to the Result section. Furthermore, we improved the overall readability.
  - E) In the Discussion, the following statement needs to be explained more fully: “Transgene expression in animal cells is typically achieved using a plasmid vector and E. coli host. Here, we showed that the Amp gene or ColE1 sequence exhibited RIGA. This finding is consistent with the ability of a plasmid bacterial sequence to silence episomal transgene expression [39}.
    - --------------Thank you for your comment. The “RIGA” in the original manuscript was a mistake, and it should have been RIGS, we apologize. Furthermore, we improved readability.
  - F) The Figure Legends must better describe the data presented, the lane designation, abbreviations, and colors.
  - -------------We have reviewed and revised the figure legends accordingly.

2. Statistical Evaluation:

- Insufficient information is presented including the number of experimental repeats, the statistical tests used, and the statistical significance of any increase or decrease in gene expression (including the modifications suggested Reviewer 2).
  - --------- We have added the statistical analyses accordingly, which appear in the newly added Figure 5 and the relevant sections in the revised manuscript, and improved overall readability.

3. Scientific Rigor

- As indicated by Reviewer 1, corrections based on the differing transfection efficiencies must be presented and discussed.
- ------------As described in the response to Reviewer 1, we did understand the reviewer’s concern (please see the response). We are prepared to modify the presentation accordingly but please further clarify your intended meaning.
- Provide the data requested on the number of repeats units introduced after transfection,
- ----------As indicated by reviewer 2, most experiments were done only once. It was because the experiments were highly time- and labor-consuming, and we could not perform multiple rounds of transfection/selection/analyses. Instead, we conducted various experiments using different sequences, which results were consistent and strengthened our argument. We understand the importance of the statistical analyses thus provided thorough statistical analyses, which appear in Fig. 5 and relevant places in the manuscript. Furthermore, we have added Fig. S2 and Fig. S3 which are composed from different transfections from Fig. 2B and Fig. 3A, and further supported the reproducibility of the result.
- Provide the clarification of Figures 6 and 7 explained by Reviewer 2.
- ------------We have provided it accordingly. Please see the response to reviewer 2.
- Provide answers to all additional issues raised by the Reviewers.
- ------------We have answered all issues raised by the reviewers. Please see the response to reviewer’s comment.
- Provide all raw data or links to the raw data as given in the Author guidelines.
- ----------All raw data were provided in the manuscript.

If you would like to make changes to your financial disclosure, please include your updated statement in your cover letter.
--------------We would like to add the following financial disclosure, “This research was supported by AMED under Grant Number JP19ae0101054”, and we will noted in our resubmission letter.

**Reviewer #1**: The manuscript presents an interesting set of observations and indicates that certain sequences, when repeated, may induce silencing (RIGS) or activate gene expression (RIGA). This type of phenomenon have been known from past studies, however, this work associates gene expression activation with potential replicative properties of the sequences.
Results, although interestinｓg, are not supported by appropriate controls and therefore I found some of the conclusions made by the authors over interpretation of the presented data.

First, experiments are based on transfection and a careful control of transfection efficiency should be presented.

--------------Thank you for your comment and assessment. We apologize, but we don’t understand the intend of your common. We have presented the colony number in the original Fig. 6A (new Figure 7A), reflecting the transfection efficiency. When we compare pKV, pDBM-d2EGFP and +B-3-31, the colony number was the lowest in pKV and highest in +B-3-31 and the medium in pDBM-d2EGFP, which was reproducible among experiments 1, 2 and 3. We have published similar experimental design, e.g. transfection of plasmid with/without IR/MAR, analysis of amplification by FISH and expression by flow cytometry, in previous papers, but we have not received similar comment before. If the reviewer may kindly suggest how we shall treat and evaluate the efficiency, we would be happy to do so.

In addition, it is not clear how many copies of the initially transfected plasmids are in fact integrated into the genome and how many (if any are in the episomal state). This could be determined by appropriate qPCR approaches.

------------Thank you for your comment; we understand the reviewer’s concern. However, our previous work had repeatedly shown that the FISH result of the stable transformants was proportional to the copy number in cells, which was determined using qPCR (PLOS ONE e52990, PLOS ONE e0175585, J. Cell Biochem. 2007) or southern blotting (Cancer Res., 2003); therefore, the IR/MAR plasmid was not maintained as an episome that was not visible in FISH, at least in the stable transformant. In the current study, the introduced gene was certainly amplified in all cases (Fig. 7), thus gene expression from the amplified structure was evaluated. We agree that it is better to present the data as gene expression per gene copy; however, evaluation of the copy number in cells requires large amount of additional experiments, which can be done in future studies, and we to publish in a timely manner. I am retiring from the University on next March and thus preparing to close my lab and my life-long research; therefore, we cannot conduct additional experiments. We think that the current paper already include sufficient work that support timey publication.

Finally, all expression determinations are made with a GFP encoding construct under CMV promoter. If this determinations of RIGS and RIGA could address potentially efficiency of recombinant protein production in the cells, a set of different promoters frequently used in mammalian expression systems should be tested to demonstrate universality of the observations.

-------------The degree of RIGS and/or RIGA may different between different promoters. Our data suggested that the prokaryotic sequences tend to be silenced by RIGS, and the viral CMV promoter may be less active in an amplified repeat. We have previously shown that the extent of silencing was highest in the CMV promoter, lowest in EF1a and moderate in the CAG promoter among the IR/MAR amplified structure (PLos ONE e41787). Our collaboration company is currently examining the application of RIGS/RIGA phenomenon in combination with R/MAR amplification in protein production. Such study may reveal the promoter selectivity. To examine the promoter dependency, additional experiments are required, and as noted above, we require timey publication, and thus, due to time restriction, we are unable to conduct further experimentation. Again, we think that the current paper already include sufficient work that support timey publication.

Minor issues:
The manuscript is well written and presented, however, parts of the introduction are in fact description of the methods/results (page). This should be replace with more general presentation and importance of the subject.

----------------Thank you for your comment. The text were originally at the Methods section and was moved to Introduction by an English editor. I completely agree with the reviewer, and have returned the text to the adequate position.

**Reviewer #2**: In the MS entitled “Repeat induces not only gene silencing (RIGS), but also gene activation (RIGA) in mammalian cells” Ogaki developed an assay system for evaluating the influence of repeat sequences on gene expression, based on in vitro ligation followed by the previously published IR (Initiation Region)/ MAR (Matrix Attachment Region) gene amplification technology in mammalian cell. This technology is use to generate high protein expression in mammalian cells. The novel contribution of the study is the analysis of human genomic repeats (e.g. LINEs, Alus), some other no specified repeats found in the genome named (B-3-31) that enhanced gene expression from tandemly amplified repeats and some core sequences from different initiation regions that the authors called C12, D8, and G5. The Shimizu lab has a great deal of experience using the IR/MAR technology applied in this study. The author’s results, therefore, highlights the potential of repeat sequences may downregulate (RIGS) or upregulate (RIGA) the expression of eGFP using the IR/MAR system. The manuscript is well written and it fits well with the scope of the journal. After the revisions have been done, I recommend publishing this article.
I have read this paper several times and submit these recommendations to you:
Results:
It will be helpful if Figure 1D it is described in more detail in the figure legend. Please add sizes of the band for each gel and define the abbreviation M and L in the figure legend.

-----------------------Thank you for your comment and we apologize for this mistake. We have now added the description to the legend of Fig. 1. Furthermore, according to the kind suggestion, we now added the band size to Fig. 1D.

For Figure 2, add a graph that summarizes the % eGFP cells and the fold effects of significant differences using the mean. The way the data is shown and the description in the results and figure legend implies that the experiments were done only one time (n=1). Do this for A, B, C, D. Please add statistical analysis to the graphs.
Figure 3 and 4 do the same as described for Figure 2.
For Figure 5C and 5D do the same as described for Figure 2.

----------------------Most experiments were done only once, since the experiments were highly time and labor-consuming, and we could not perform multiple rounds of transfection/selection/analyses that support the statistical analyses. Instead, we conducted experiments using different sequences, which results were consistent and strengthen the argument. We agree that the statistical evaluation is important, but it was impossible from a single result set. However, we conducted multiple rounds of independent transfections using B-3-31 repeat and G5 repeat; therefore, we could plot graphs and evaluate the statistical significance for the effect of addition of such repeat. Furthermore, we analyzed 6 different kinds of sequences that showed RIGA and 6 different kinds of sequences that showed RIGS. We then plotted graphs and statistically analyzed the significant differences between these two groups. The results of these analyses are shown in the newly added Fig. 5. The statistical analyses supported our arguments.

For Figure 6A the figure legend describes “the colony number per dish was counted and plotted; a small and large colony was defined as a colony comprising less than or more than 100 cells, respectively”. The manuscript does not explain why the size is important. Please explain why the data was presented this way. Is there an actual physical measure you used to determine 100 cells? If so, please add to the figure legend.

----------------Thank you for your suggestion. Indeed, the number of small colonies had an important implication. We have included a description in lines 510-514, to further explain it.

Figure 7B 3rd graph showing RIGS looks the same as baseline pBM-d2EGFP. Please shoe the % of the DMs. This data does not support that RIGS result in “preferential amplification at the extrachromosomal DMs” as stated in the results. Especially when you compared the baseline pBM-d2EGFP from the RIGA experiments.

------------------pBM-d2EGFP was amplified to generate a silent structure, by the RIGS. Therefore, the consequence would be the same between pBM-d2EGFP alone and the co-transfection of the plasmid and the RIGS repeat. However, we appreciate the comment from the reviewer, and have deleted the text suggesting that the RIGS favored extrachromosomal amplification.

While revising your submission, please upload your figure files to the Preflight Analysis and Conversion Engine (PACE) digital diagnostic tool, [https://pacev2.apexcovantage.com/](https://jpn01.safelinks.protection.outlook.com/?url=https%3A%2F%2Fpacev2.apexcovantage.com%2F&data=02%7C01%7Cshimizu%40hiroshima-u.ac.jp%7C60e4d632881842ecb97d08d7ea8828d9%7Cc40454ddb2634926868d8e12640d3750%7C1%7C1%7C637235742835822911&sdata=2n9NhpTYrSiZ0jwTXS%2FYQGTYpQ6DQYlwzzOucm6mmRk%3D&reserved=0). PACE helps ensure that figures meet PLOS requirements. To use PACE, you must first register as a user. Registration is free. Then, login and navigate to the UPLOAD tab, where you will find detailed instructions on how to use the tool. If you encounter any issues or have any questions when using PACE, please email us at [figures@plos.org](mailto:figures@plos.org). Please note that Supporting Information files do not need this step.
